# Therapeutic potential of a novel prodrug of green tea extract in induction of apoptosis via ERK/JNK and Akt signaling pathway in human endometrial cancer

**DOI:** 10.1186/s12885-020-07455-3

**Published:** 2020-10-06

**Authors:** Gene Chi Wai Man, Jianzhang Wang, Yi Song, Jack Ho Wong, Yu Zhao, Tat San Lau, Kam Tong Leung, Tak Hang Chan, Huating Wang, Joseph Kwong, Tzi Bun Ng, Chi Chiu Wang

**Affiliations:** 1 Department of Orthopaedics and Traumatology, Faculty of Medicine, The Chinese University of Hong Kong, Prince of Wales Hospital, Shatin, Hong Kong SAR China; 2 Department of Obstetrics and Gynaecology, Faculty of Medicine, The Chinese University of Hong Kong, Prince of Wales Hospital, Shatin, Hong Kong SAR China; 3 Department of Gynecology and Obstetrics, Women’s Hospital, School of Medicine, Zhejiang University, Hangzhou, China; 4 School of Biomedical Sciences, Faculty of Medicine, The Chinese University of Hong Kong, Shatin, Hong Kong SAR China; 5 Department of Pediatrics, Faculty of Medicine, The Chinese University of Hong Kong, Prince of Wales Hospital, Shatin, Hong Kong SAR China; 6 Department of Applied Biology and Chemical Technology and State Key Laboratory of Chemical Biology and Drug Discovery, The Hong Kong Polytechnic University, Hung Hom, Hong Kong SAR China; 7 Li Ka Shing Institute of Health Sciences, Faculty of Medicine, The Chinese University of Hong Kong, Shatin, Hong Kong SAR China

**Keywords:** ProEGCG, Endometrial cancer, Apoptosis, Anticancer, Anti-angiogenesis, Akt pathway

## Abstract

**Background:**

Previous studies have shown a major green tea polyphenol (−)-epigallocatechin-3-gallate ((−)-EGCG) as a powerful anti-cancer agent. However, its poor bioavailability and requirement of a high dosage to manifest activity have restricted its clinical application. Recently, our team synthesized a peracetate-protected derivative of EGCG, which can act as a prodrug of (−)-EGCG (ProEGCG) with enhanced stability and improved bioavailability in vitro and in vivo. Herein, we tested the therapeutic efficacy of this novel ProEGCG, in comparison to EGCG, toward human endometrial cancer (EC).

**Methods:**

In this study, the effects of ProEGCG and EGCG treatments on cell growth, cell survival and modulation of intracellular signaling pathways in RL95–2 and AN3 CA EC cells were compared. The antiproliferative effect was evaluated by cell viability assay. Apoptosis was measured by annexin/propidium iodide staining. Expression of mitogen-activated protein kinases, markers of proliferation and apoptosis were measured by immunoblot analysis. In addition, the effects of ProEGCG and EGCG on tumor growth, vessel formation and gene expression profiles on xenograft models of the EC cells were investigated.

**Results:**

We found that treatment with ProEGCG, but not EGCG, inhibited, in a time- and dose-dependent manner, the proliferation and increased apoptosis of EC cells. Treatment with low-dose ProEGCG significantly enhanced phosphorylation of JNK and p38 MAPK and inhibited phosphorylation of Akt and ERK which are critical mediators of apoptosis. ProEGCG, but not EGCG, elicited a significant decrease in the growth of the EC xenografts, promoted apoptotic activity of tumour cells in the EC xenografts, and decreased microvessel formation, by differentially suppressing anti-apoptotic molecules, NOD1 and NAIP. Notably, no obvious adverse effects were detected.

**Conclusions:**

Taken together, ProEGCG at a low dose exhibited anticancer activity in EC cells through its anti-proliferative, pro-apoptotic and anti-tumor actions on endometrial cancer in vitro and in vivo. In contrast, a low dose of EGCG did not bring about similar effects. Importantly, our data demonstrated the efficacy and safety of ProEGCG which manifests the potential of a novel anticancer agent for the management of endometrial cancer.

## Background

Endometrial cancer (EC) is one of the most common gynecological malignancies in the developed world. It ranked as the sixth most common cause of death from cancer which only affects women, and the 15th most commonly occurring cancer overall [1]. Although the majority of ECs are diagnosed and can be treated when confined to the uterus, recurrence has been reported in up to 25% of the cases. The response rate of advanced and metastatic cancer to standard chemotherapy is typically low, and consequently patient prognosis remains poor. Given the escalating incidence of this cancer and the paucity of effective treatments for advanced EC, it is imperative to search for novel agents for the effective management of this cancer.

**Fig. 1:**
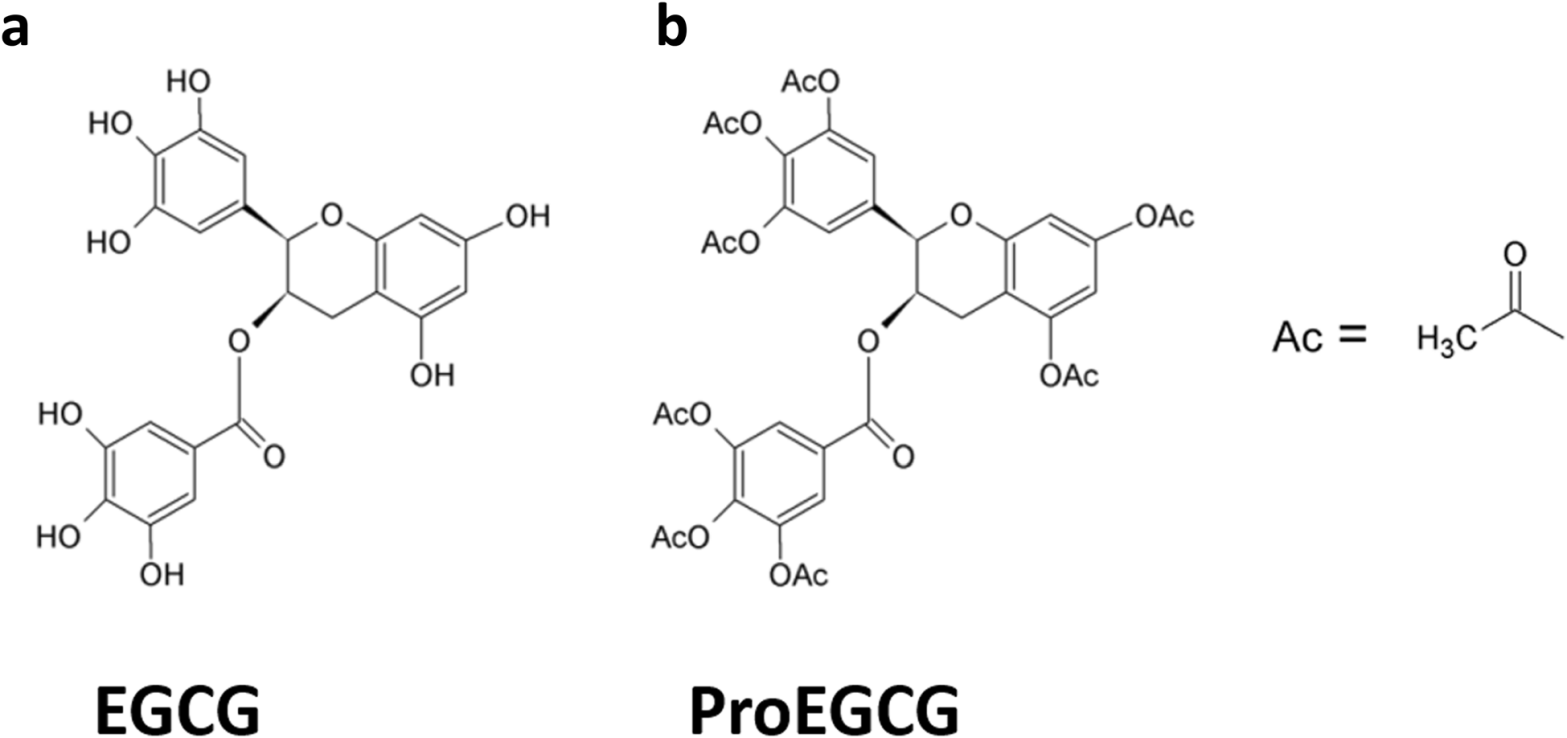
Molecular structures of EGCG (**a**) and ProEGCG (**b**)

Tea is the second most popular beverage in the world after water. The polyphenol found in green tea, (−)-epigallocatechin-3-gallate (EGCG), exhibits potent anti-oxidative, anti-mitotic and anti-angiogenic properties [2–4]. EGCG has long been known to inhibit carcinogenesis of diverse tumor types [5–7]. It acts through inhibiting a variety of cancer-related pathways, including cell proliferation and tumor growth, invasion and metastasis, and angiogenesis, and to induce apoptosis and cell cycle arrest in transformed cells. EGCG is devoid of significant untoward side-effects and toxicity, and thus has gained considerable attraction as a potential therapeutic in clinical scenarios. However, the bulk of these studies employed an exceedingly high dosage which is pharmacologically infeasible [3, 8, 9]. In addition, EGCG is notoriously unstable and has the shortcoming of poor bioavailability [10]. Hence, in recent years, our team synthesized a peracetate-protected derivative of EGCG, produced by acetylation of EGCG, which can act as a prodrug of EGCG (ProEGCG) with enhanced stability and improved bioavailability in vitro and in vivo [11] (Fig. 1).

The effectiveness of ProEGCG on breast cancer cells and in breast cancer–bearing nude mice was proven by the elevated levels of proteasome inhibition, growth suppression, and induction of apoptosis [12–14]. In addition, ProEGCG with anti-oxidative and anti-angiogenic activities significantly thwarted the development, growth and angiogenesis of experimental endometriosis in mice with high efficacy and good bioavailability [15]. Recently, we demonstrated that ProEGCG not only downregulated vascular endothelial growth factors (VEGFs) but also inhibited tumor-associated macrophage (TAM)-secreted VEGFA in EC [16].

Despite the efficacy of ProEGCG in suppressing cancer growth, its therapeutic effect and underlying mechanism on EC cells remain to be elucidated. Hence, in this study, we investigated the anticancer effect and the related mechanisms of ProEGCG toward EC cells. Additionally, in order to acquire more knowledge necessary for the design of drugs for preclinical usage, we explored the in vivo therapeutic effect of ProEGCG by using two subcutaneous xenograft models of endometrial cancer.

## Methods

### Reagents and chemicals

HPLC-grade (−)-Epigallocatechin-3-gallate (EGCG, 99% purity), dimethyl sulfoxide (DMSO), and (3-(4,5-dimethylthiazol-2-yl)-2,5-diphenyltetrazolium bromide) (MTT) were purchased from Sigma-Aldrich. Dulbecco’s modified Eagle’s Medium, α-modified Eagle’s Medium and 0.5% trypsin-5.3 mm EDTA solution, certified fetal bovine serum (FBS)-heat inactivated and penicillin–streptomycin–neomycin antibiotic mixture was obtained from Thermo Fisher Scientific (Burlington, Canada). Amersham ECL Western blotting detection reagents were purchased from GE Healthcare Biosciences (Pittsburgh, PA).

### ProEGCG synthesis

ProEGCG (EGCG octaacetate) (purity ≥98%) was synthesized from (−)-EGCG by acetylation of the reactive hydroxyl groups according to our published procedure [11]. Purity was determined by by liquid chromatography. For all experiments, EGCG and ProEGCG were always freshly prepared in a DMSO stock solution (1000×) and the solution was directly diluted to a desired concentration with the culture medium so that the final concentration of DMSO did not exceed 0.1%.

### Cell lines

Two human EC cell lines, AN3 CA (Cat. No. ATCC® HTB111) and RL95–2 (Cat. No. ATCC® CRL-1671) were purchased from American Type Culture Collection (ATCC) while a normal human endometrial epithelial cell (HEECs) cell line was bought from Wuhan PriCells Biomedical Technology (China). AN3CA cells were grown in α-EMEM supplemented with 10% FBS and 1% PSN, while RL95–2 and HEECs were cultured in DMEM/F12 supplemented with 10% FBS and 1% PSN, in an atmosphere of 5% CO_2_ at 37 °C.

### Cell proliferation assay

Cell proliferation was measured using the 3-(4,5-dimethylthiazol-2-yl)-2,5-diphenyl tetrazolium (MTT) (Sigma-Aldrich) dye reduction method. In brief, the cells were plated at a density of 10,000 cells/well in a 96-well plate and cultured in the respective culture medium. After incubation for 24 h, the cells were treated with various (20 μM – 60 μM concentrations) of either EGCG or ProEGCG. Cells treated with 0.1% DMSO were used as control. The medium was renewed daily. Cell viability was determined at 24, 48, and 72 h by monitoring the changes of absorbance using a microplate reader (Spectramax, Gemini) at the wavelength of 570 nm. The percentage of survival cells was calculated as the ratio (A_570_) of the absorbance of treated cells over the absorbance of control cells. In each of the experiments, triplicates were set up for each drug concentration (*n* = 5), and assays were repeated in three independent experiments.

### 3D culture

EC cells (AN3CA: 10,000 cells. RL95–2: 20,000 cells) were mixed with 500 μl extracellular matrix (ECM; Matrigel, BD Biosciences) and loaded into each well of the chamber slide (Nunc Lab-Tek, Rochester, NY, USA). After the gel had solidified, EC cells were seeded on top of the gel, and treated with serial concentrations of either EGCG (20, 40, and 60 μM) or ProEGCG (20, 40, and 60 μM) for 5 days. In order to test the capability of ProEGCG to reduce cell growth, the 3D monocultures were allowed to form till day 7, prior to administering either EGCG treatment or ProEGCG treatment for 5 days.

### Flow cytometric detection of apoptosis

In the apoptosis assay, tumour cells (2 × 10^5^ cells/ml) were seeded in 6-well plates, and then treated with either EGCG or ProEGCG for 48 h and 72 h. To analyze apoptotic activity, floating and attached cells were collected and analysed by double staining with FITC-conjugated Annexin V and propidium iodide (PI) according to the manufacturer’s instructions (Annexin V-FITC Apoptosis Staining / Detection Kit, Abcam). The percentage of apoptotic cells was analysed using a Cytomics FC500 flow cytometer (Beckman Coulter) and Cell-Quest software. For each sample, data from 10,000 cells were recorded.

### Western blot analysis

Western blotting analysis was performed according to our previous protocol [16]. In brief, the tumour cells were grown to ~ 80% confluency in 100- mm dishes, and then treated with either EGCG or ProEGCG for 72 h at 20, 40, and 60 μM concentration, with daily renewal of medium with EGCG (or ProEGCG). The treated and control cells were rinsed with PBS and harvested using lysis buffer (50 mM Tris–HCl, 150 mM NaCl, 1% Nonidet, 0.5% sodium deoxycholate, 0.1% sodium dodecyl sulfate, and 1 μM sodium orthovanadate) supplemented with protease inhibitor cocktail (Sigma-Aldrich). Protein concentrations were quantified using the Bio-Rad protein assay kit. Equivalent amounts of total protein (30 μg) were separated by employing 8 ~ 15% SDS–polyacrylamide gel electrophoresis, transferred to a polyvinylidene difluoride membrane, and subjected to Western blot analysis for probing with primary antibodies (Akt1/2/3 (H-136), p-Akt1/2/3 (Ser-473), ERK1 (K-23), p-ERK (E-4), JNK (FL), p-JNK (G-7), p38α (C-20), p-p38 (D-8); GAPDH (FL-335) from Santa Cruz Biotechnology Inc.; cleaved caspase-3 (Asp175) and cleaved PARP (Asp214) antibodies from Cell Signalling Technology; caspase-3 (E-87) and CD34 (EPR2999) were obtained from Abcam). Then, the membrane was incubated with appropriate horseradish peroxidase conjugated secondary antibody and visualized with the Amersham ECL Western blotting detection kit (GE Healthcare Biosciences). Experiments were repeated in three independent experiments.

### In vivo tumor xenografts

Fifteen female athymic nude mice (nu/nu) (5–6 weeks old, 16-20 g) were obtained from the Laboratory Animal Services Centre of the Chinese University of Hong Kong. The animals were kept in wire cages in laminar air flow cabinets at a temperature of 20–22 °C, a humidity of 30–50%, and a 12-h/12-h light/dark cycle. Autoclaved chow pellets and autoclaved tap water were provided ad libitum. The animal colonies were maintained in a specific pathogen-free environment with regular screenings for detecting the presence of murine pathogens. The experimental protocol was conducted under the license from the Hong Kong Department of Health and with approval from the Chinese University of Hong Kong Animal Research Ethics Committee. Endometrial xenografts were established by implanting suspensions of endometrial tumor cells, as previously described [16]. In brief, mice were anesthetized in a chamber prefilled with 1.5% isoflurane in oxygen. Endometrial xenografts were then established by implanting suspensions of endometrial tumour cells (1 × 10^6^ cells resuspended in 50 uL of HBSS-buffer mixed with 50/50 (v/v) with Matrigel (BD Biosciences, San Jose, CA)) subcutaneously into the right flanks of the mice. Tumor growth was monitored weekly, and tumor volumes (mm^3^) were measured with an electronic caliper, using the following formula: tumor volume (mm^3^) = 0.5 x length (mm) x width^2^ (mm^2^). After 2 weeks, when the size of the tumor lesion reached 100 mm^3^, the mice were randomized into four groups with the first group without treatment, and the remaining three groups treated by oral gavage daily with (group 2) ProEGCG (50 mg/kg), (group 3) EGCG (50 mg/kg) or (group 4) vehicle (olive oil) (*n* = 5 for each group). Body weight and the tumours were regularly monitored. At the end of the experiment, mice were sacrificed by using an overdose of non-painful anaesthesia (Ketamine/Xylazine cocktail). Macroscopic lesions were recorded and metastatic tumor nodules were dissected, weighed and paraffin-embedded for further immunohistochemical staining. In addition, blood was taken for biochemical analyses (creatinine, alanine transaminase (ALT) and alkaline phosphatase (ALP); Abcam). Based on our previous publication, plasma levels of ProEGCG can be maintained at 1.36 μg/mL for 360 min after an intraorbital intravenous injection at 50 mg/kg in mice [17]. In addition, the dosage was determined according to our previous publication [16].

### Apoptosis microarray

Total RNA in the endometrial tumour xenograft samples was extracted by using RNeasy mini Plus kit (Qiagen). To ascertain the apoptosis-related molecules regulated by ProEGCG treatment, gene expression of the xenograft tissues was examined by angiogenesis microarray. A microarray experiment was performed following previous procedures [18]. Data were acquired by Feature Extraction 10.5 and analysed by GeneSpring GX software (Agilent). Likewise, the microarray findings were further validated by using quantitative real-time RT-PCR, based on previous experiments [18]. TaqMan probes of human NOD1 (Hs01036720_m1), NAIP (Hs03037952_m1), BAG1 (Hs00185390_m1) and GAPDH (Hs02758991_g1) were used and purchased from Thermos Life Technologies.

### Histological analysis

Tumour tissues were fixed in 10% buffered formalin overnight and embedded in paraffin. The blocks were cut into 4- μm micrometer thick slices for different staining. Sections were stained with haemotoxylin and eosin (HE) for morphological visualization. For immunohistochemistry, sections were incubated with the appropriate dilution of primary antibodies overnight, followed by 3,3-diaminobenzidine (DAB) staining. Terminal deoxynucleotidyl transferase-mediated dUTP nick end labelling (TUNEL) staining was performed using the ApopTag® peroxidase in situ apoptosis detection kit (Milipore) according to the manufacturer’s instructions. After mounting, images were captured with a Leica DM2500 microscope for further processing using ImageJ (Bethesda). Slides of endometrial cancer tissue samples from the vehicle-treated xenograft were collected and incubated with a brief treatment of DNAse 1 after permeabilization were used as the positive control of the TUNEL assay. Slides treated with isotype-matched IgG but without each primary antibody served as the negative control.

### Statistical analysis

Statistical analysis was performed using the SPSS statistics software (IBM Corp.). All results are presented as mean ± standard error of the mean (S.E.M) from at least three independent experiments. Student’s *t*-tests were performed to analyse the differences between two groups. One-way analysis of variance (ANOVA) followed by post hoc comparisons of individual groups using Bonferroni correction was used for comparisons between multiple groups. In the event of an absence of normality of distribution, nonparametric Kruskal-Wallis and Wilcoxon tests were used where appropriate. Differences were considered significant when *P* < 0.05.

## Results

### ProEGCG exerts an anti-proliferative effect in vitro on human EC cell lines

**Fig. 2:**
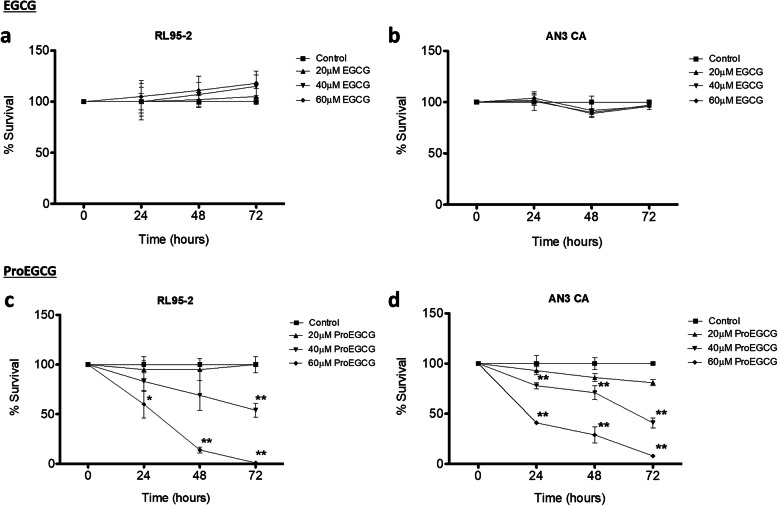
Effects of ProEGCG and EGCG on cell proliferation of human endometrial cancer cells. RL95–2 and AN3 CA cells were treated with increasing doses of (**a**, **b**) EGCG (20, 40, and 60 μM) and (**c**, **d**) ProEGCG (20, 40, and 60 μM) for 24, 48 and 72 h. Cell viability was assessed by MTT assay. The percentage of viable cells was calculated as the ratio of the number of treated cells to the number of control cells. Data are presented as mean ± S.E.M. Significant differences from control: *P < 0.05 and **P < 0.01

The anti-proliferative effects of different concentrations of ProEGCG and EGCG on two EC cell lines, RL95–2 and AN3 CA, were compared. ProEGCG lowered the viability of the two EC cells in a dose- and time-dependent manner (Fig. 2). In both RL95–2 and AN3 CA cells, ProEGCG significantly decreased cell proliferation at 40 μM and 60 μM concentrations after incubation for 48 h (*P* < 0.05). Over 90% of both RL95–2 and AN3 CA endometrial cells became nonviable following exposure to 60 μM ProEGCG for 72 h (*P* < 0.01). In contrast, EGCG failed to elicit a significant reduction in the growth of both EC cells (Fig. 2a and b). The IC50 value of ProEGCG in RL95–2 cells was found to be 43.7 μM and 38.0 μM after 48 h and 72 h (Fig. 2c), respectively, whereas the IC50 value of ProEGCG in AN3 CA cells was 48.8 μM and 34.7 μM after 48 h and 72 h (Fig. 2d), respectively. On the other hand, the profile of cytotoxicity of ProEGCG to normal endometrial cells (HEEC cells) revealed an IC50 value that exceeded 352 μM, suggesting that ProEGCG was nontoxic to normal cells (Supplementary Fig. 1). In addition, treatment with ProEGCG also significantly abolished colony formation in the 3D monocultures of metastatic EC cells when treated from day 2 to day 7 (Supplementary Fig. 2A) or when treated after 1 week (Supplementary Fig. 2B).

### ProEGCG induces EC cell death via caspase dependent apoptosis

**Fig. 3:**
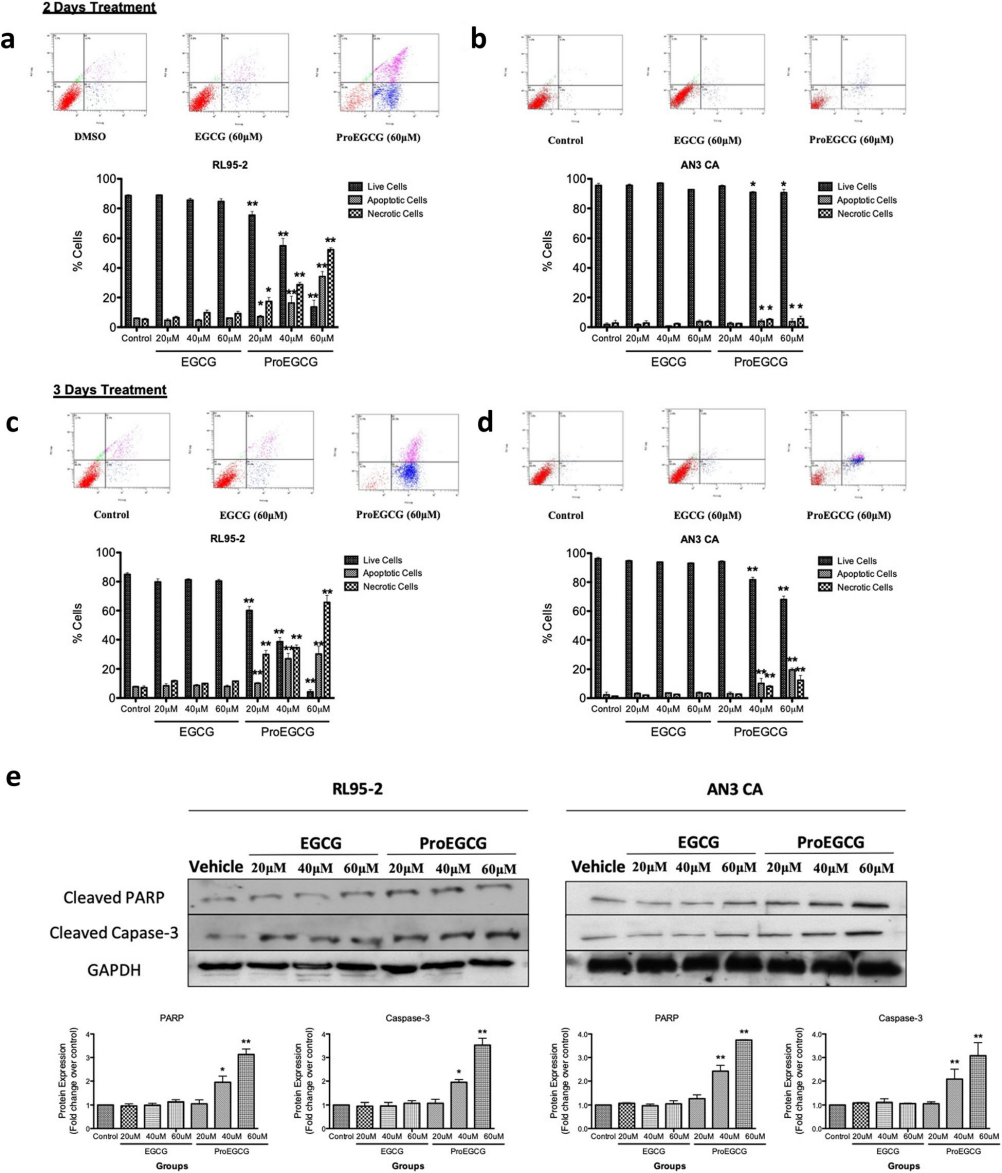
ProEGCG induced apoptosis in RL95–2 and AN3 CA cells in a time-dependent manner. Cells were treated with increasing concentrations of EGCG and ProEGCG (**a**) RL95–2 cells and (**b**) AN3 CA cells for 48 h and (**c**) RL95–2 cells and (**d**) AN3 CA cells for 72 h. Apoptotic cells were quantitatively analyzed by flow cytometry with annexin V-FITC and propidium iodide staining. **e** Lysates from cells treated with ProEGCG and EGCG were assayed for PARP and caspase-3 cleavage by Western blotting. GAPDH was used as a loading control. Data are presented as mean ± S.E.M. Significant differences from control: *P < 0.05 and **P < 0.01

The effects of ProEGCG and EGCG on the induction of apoptosis in EC cells were assessed by FACS assay. A significant induction of apoptosis was observed at all-time intervals after treatment of both EC cell lines with ProEGCG (Fig. 3a and b). However, EGCG did not bring about any significant inhibition of the growth of endometrial cells (Fig. 3a and b). This result was further verified by immunocytostaining of the EC cells with Annexin V and PI after treatment with ProEGCG (Supplementary Fig. 3). The data confirmed apoptotic activity in the EC cells upon treatment with 40 and 60 μM ProEGCG. Moreover, apoptosis was further detected in both cell lines following treatment with ProEGCG and EGCG by the presence of apoptotic markers, cleaved caspase-3 and cleaved PARP, using Western blot analysis. ProEGCG upregulated the expression of cleaved caspase-3 and cleaved PARP in a dose-dependent manner (Fig. 3c). The apoptotic effect was found to be most potent at 60 μM as more intense cleaved caspase-3 and cleaved PARP bands were observed.

### ProEGCG induces JNK and p38 MAPK phosphorylation and suppresses Akt and ERK signaling in EC cells

**Fig. 4:**
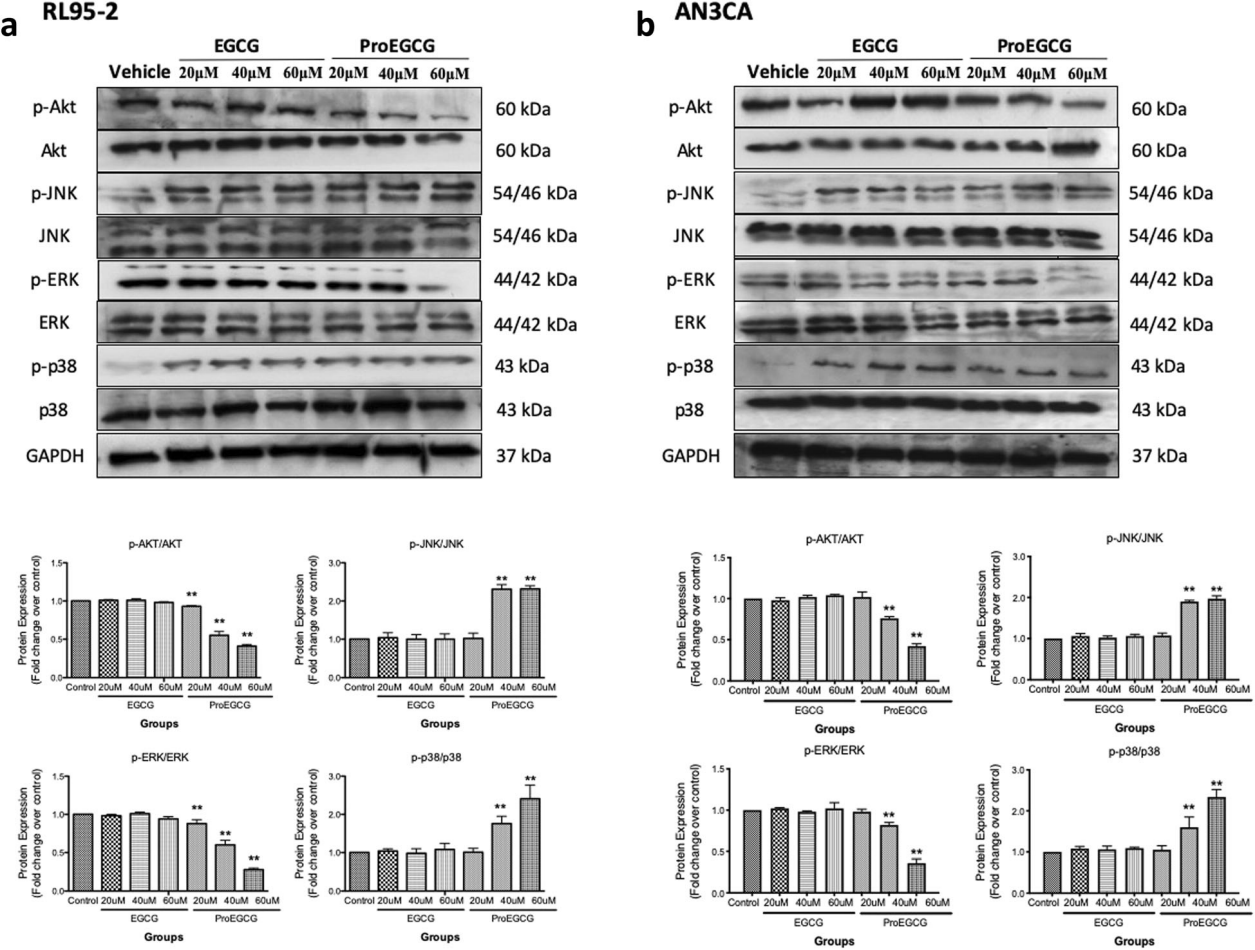
Effects of ProEGCG and EGCG on activating signaling Akt and MAPKs signaling pathway. Effects of ProEGCG and EGCG on the activation of Akt, ERK, JNK and p-38 in human endometrial (**a**) RL95–2 cells and (**b**) AN3 CA cells by Western blotting. Representative Western blot of results from 3 independent experiments are shown in upper panels. Quantification of protein expressions were measured. Full-length of the gels are presented in Supplementary Fig. 5. Data are presented as mean ± S.E.M. Significant differences from control: *P < 0.05 and **P < 0.01

To further investigate the anticancer mechanism of the MAPK signaling pathway in EC cells by ProEGCG treatment, in vitro cultures of human EC cells were performed. The results showed that an increased concentration of ProEGCG treatment significantly stimulated the increased phosphorylation of JNK and p38 MAPK (Fig. 4). In contrast, a significant reduction of phosphorylation of ERK in both EC cells after treatment with ProEGCG for 72 h was observed (Fig. 4). The decline in phosphorylation was most remarkable when EC cells were treated with 60 μM ProEGCG. Likewise, there was a similar reduction of phosphorylation of Akt in both EC cells after 72 h of treatment with ProEGCG (Fig. 4). However, these changes were not observed in the EC cells treated with various concentrations of EGCG.

### ProEGCG reduces tumor growth in vivo

**Fig. 5:**
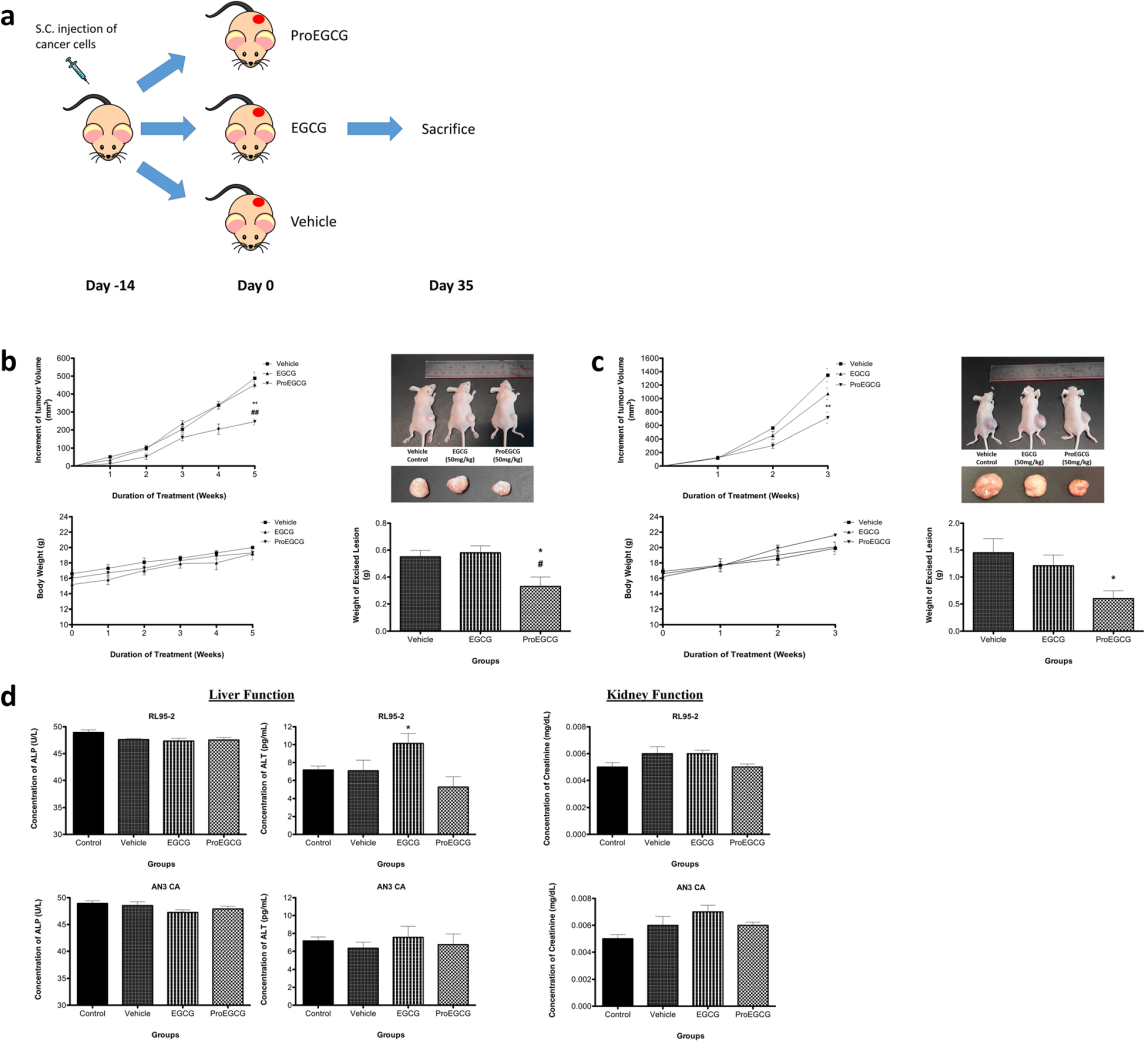
ProEGCG inhibits proliferation of endometrial carcinoma in vivo. **a** Schematic establishment of the xenograft model in this study. **b** Rl95–2 and (**c**) AN3 CA cells were injected subcutaneously into athymic nude mice. Tumors were allowed to grow to a size around 100 mm^3^ and were then orally treated with ProEGCG (50 mg/kg), EGCG (50 mg/kg) or vehicle (olive oil) (*n* = 5 for each group). The increment of tumor volume and changes of body weight were assessed longitudinally. **d** Evaluation of hemato-biochemical markers from plasma samples of tumor-bearing mice after treatment with ProEGCG. Full-length of the gels are presented in Supplementary Fig. 5. Data are presented as mean ± S.E.M. Significant differences from control: *P < 0.05 and **P < 0.01. ALP, alkaline phosphatase; ALT, alanine aminotransferase

Subcutaneous tumor xenografts, developed from two human EC cell lines (RL95–2 and AN3 CA), were successfully established individually. ProEGCG elicited significant growth inhibition of the lesion in the endometrial tumor xenograft model. In the RL95–2 xenograft model, tumor size was significantly reduced after 5 weeks of treatment with ProEGCG (Fig. 5a). The tumor mass (g) was significantly reduced in mice treated with ProEGCG (vehicle group: 0.55 ± 0.11 vs ProEGCG group: 0.33 ± 0.16; *P* ≤ 0.05) (Fig. 5a). However, there was no significant reduction in tumor mass in mice treated with EGCG. In the AN3 CA xenograft model, the tumour size was significantly reduced after oral administration of ProEGCG for 3 weeks (Fig. 5b). This was further confirmed by the reduction in tumour mass (g) ex vivo (vehicle group: 1.45 ± 0.59 vs ProEGCG group: 0.60 ± 0.32; *P* ≤ 0.05) (Fig. 5b). Similarly, there was no significant reduction found in mice treated with EGCG. Notably, there was no toxicity found in both xenograft models in response to treatment with ProEGCG (Fig. 5c).

**Fig. 6:**
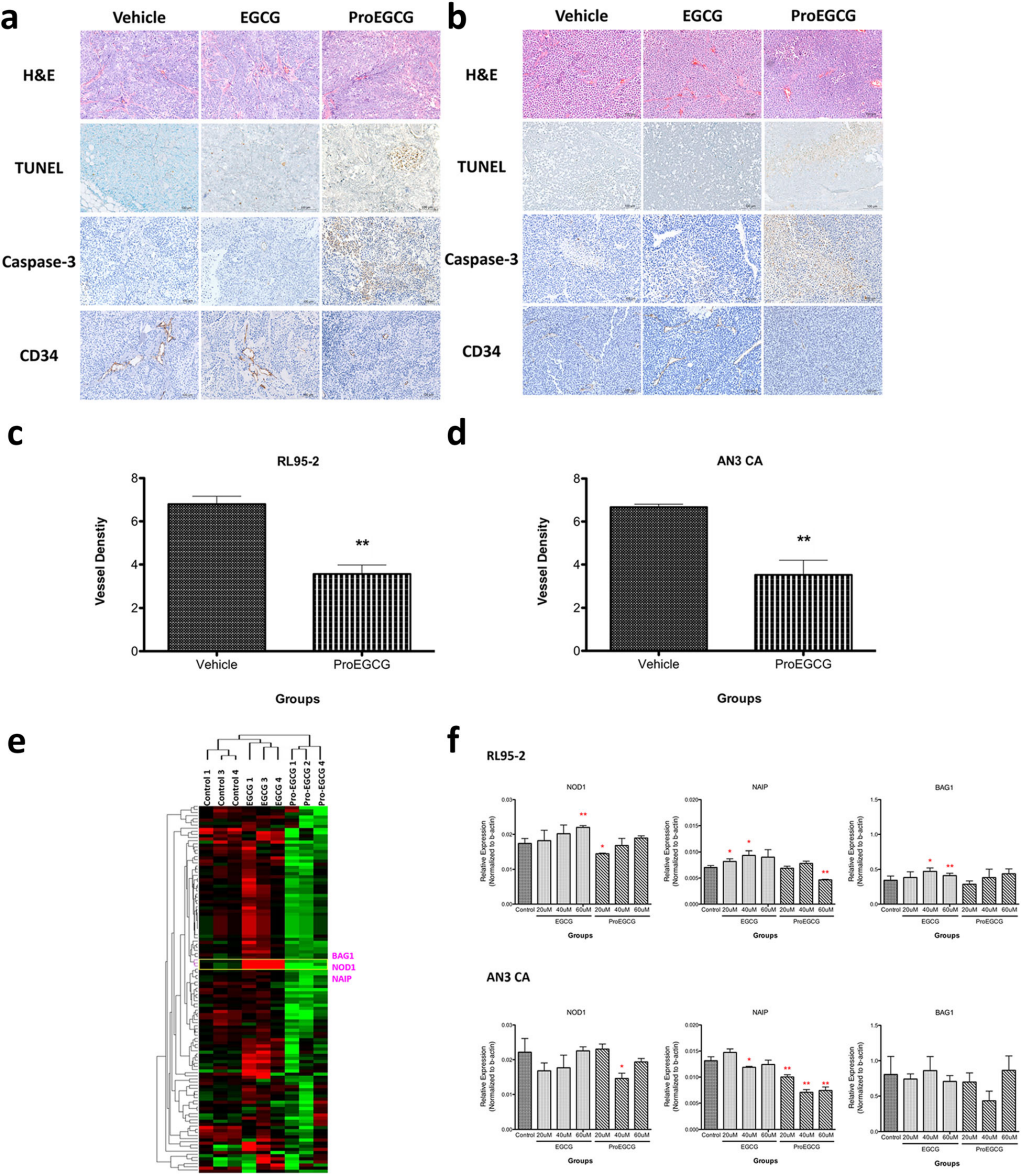
Apoptotic and microvessel development in endometrial carcinoma xenograft tumors after ProEGCG treatment. Haemotoxylin & eosin staining, TUNEL assay, caspase-3 and CD34 in (**a**) Rl95–2 and (**b**) AN3 CA xenograft tumors were analyzed by immunohistochemistry (magnification 200x). **c, d** The expression levels of CD34-postive cells were quantified. **e** Hierarchical clustering shows differential regulation of BAG1, NOD1 and NAIP (pink tree branch, yellow box) after EGCG and ProEGCG treatments in human EC cell xenografts. **f** Quantitative analysis by real-time PCR validated the differential expression of NOD1, NAIP and BAG1 after EGCG and ProEGCG treatment in human EC cell lines. Data are presented as mean ± S.E.M. Significant differences from control: *P < 0.05 and **P < 0.01

Further analysis of the excised lesion disclosed induction of apoptosis and necrosis when the mice were treated with ProEGCG (Fig. 6a and b). Similar to our previous report, treatment with ProEGCG significantly impeded angiogenesis qualitatively (Fig. 6a and b) as well as quantitatively (Fig. 6c and d) in EC xenograft models. The abated angiogenesis was further corroborated by the significant diminution in microvessel density (Fig. 6c and d). In addition, expression of genes associated with apoptosis was also examined using microarray analysis in the endometrial tumor xenograft tissues with or without ProEGCG treatment. Our findings revealed significant down-regulation of human NOD1, NAIP, and BIG1 in xenografts treated with ProEGCG compared to vehicle-treated xenografts (Fig. 6e and f).

## Discussion

In this study, we examined the growth inhibitory effect of ProEGCG on EC cells both in vitro and in vivo. The results indicated that ProEGCG decreased the viability of human EC cells in a dose-dependent manner. In addition, ProEGCG induced caspase-3 expression which subsequently upregulated cleaved PARP and triggered apoptosis. Treatment of EC cells with ProEGCG significantly enhanced the phosphorylation of JNK and p38 MAPK and inhibited the phosphorylation of Akt and ERK which are critical mediators of apoptosis. Consistent with the in vitro findings, the therapeutic effects of ProEGCG in the EC xenografts were revealed in a significantly smaller tumour volume together with enhanced apoptotic activity and suppressed microvessel formation.

Green tea is one of the most popular beverages consumed around the world. Among the many polyphenolic compounds, EGCG has been found to be the most effective chemopreventive agent. A sizeable number of studies have shown that EGCG could suppress cell proliferation and induce apoptosis in many types of cancer cells [19–21]. However, EGCG has a low bioavailability in vivo [10]. In order to uplift the stability of EGCG, we synthesized a peracetate-protected EGCG (ProEGCG) by modifying the reactive hydroxyl groups with peracetate groups. The resulting ProEGCG was six fold more stable than EGCG and it demonstrated an augmented efficacy in inducing cancer cell death [11]. Likewise, the tumor volume was significantly curtailed in xenograft models of human breast cancer after administration of ProEGCG [13]. More recently, we noted that ProEGCG can exert an anti-angiogenic effect in human endometrial cancer xenograft models through interference with microvessel formation [16]. In this study, we further demonstrated the anticancer effect of ProEGCG on endometrial cancer cells in vitro and in vivo.

Apoptosis is a well-known cellular defence mechanism that eliminates cancer cells and plays a vital role in the prevention of tumorigenesis. Many anticancer drugs work by regulating apoptosis-related signals to induce cancer cell death [22, 23]. Although a study showed EGCG can induce apoptosis in human endometrial adenocarcinoma cells, it would require a relatively high concentration of EGCG (100–150 uM) to significantly reduce the cell viability and induce apoptosis [3]. In our study, we utilized a lower concentration of EGCG (20–60 uM) to find the lack of response on the EC cells. This result actually resembled a previous finding [13]. In this study, we investigated whether ProEGCG can induce apoptosis in the human endometrial cancer cells. Annexin V/FITC and PI were used to stain the cells in order to distinguish between viable, apoptotic, and necrotic cells [23]. Our experimental results showed that apoptotic activity escalated with the concentration of ProEGCG, while the percentages of early and late apoptotic cells were also increased. While the process of apoptosis in carcinoma involves a large number of changes in the apoptotic proteins, our results showed that ProEGCG markedly induced apoptosis of EC cells by upregulation of the expression levels of cleaved caspase-3 and cleaved PARP. However, these changes were indiscernible when EC cells were treated with EGCG.

The MAPKs is a family of serine/threonine kinases involved in apoptosis and cell survival [24]. Based on the findings of Mahoher et al., EGCG inhibits cellular proliferation via inhibiting ERK activation and inducing apoptosis via ROS generation and p38 activation in endometrial carcinoma cells [3]. Owing to ProEGCG being an analogue of EGCG, we analysed whether ProEGCG has a role in affecting EC cell proliferation through the expression of MAPKs p-38, JNK and ERK in both EC cell lines. Our result showed an enhancement in phosphorylation expression of JNK and p-38 in both EC cell lines when treated with ProEGCG, which implicated the induction of apoptosis by ProEGCG in EC cells. Further, ERK activation was also downregulated, which in turn might be responsible for the undermined cellular growth and proliferation of the EC cells. With the MAPK pathway closely linked with the PI3K/Akt pathway, a previous study showed the reduction of cancer cells growth through inhibiting the PI3K/Akt/mTOR signalling pathway [25]. The PI3K/Akt/mTOR pathway is a key cellular signalling mechanism responsible for proliferation, migration, metabolism, and drug resistance [26–29]. Similarly, the activation of Akt, through a PI3K-dependent mechanism can also promote apoptosis via signal-regulating kinase 1 (ASK1)/MAPK signalling pathway [30]. According to our study, the phosphorylation of Akt was attenuated by an increase in the concentration of ProEGCG in the EC cells. Hence, a possible mechanism of ProEGCG to trigger EC cell death was to activate Akt, which further promotes several proapoptotic signalling proteins in the MAPKs families, to stimulate apoptosis. Taken together, it is suggested that ProEGCG induces rapid and transient activation of Akt, which inhibits the PI3K/Akt/mTOR signalling pathway, and MAPK signalling pathway to inhibit proliferation and promote apoptotic signal in the EC cells.

Eventually, the activation of this signalling pathway can trigger cell death by activating several proapoptotic signalling proteins, including JNK and p38 MAPK. In our study, the phosphorylation of Akt was attenuated by an increase in the concentration of ProEGCG in the EC cells. Hence, a possible mechanism of ProEGCG to trigger EC cell death was to activate Akt, which further promotes several proapoptotic signalling proteins in the MAPKs families, to stimulate apoptosis. The PI3K/Akt/mTOR pathway is a key signalling mechanism for cell survival proliferation, migration, metabolism, and drug resistance [26–29]. Taken together, the evidence suggest that ProEGCG induces rapid and transient activation of Akt, which in turn inhibits the PI3K/Akt/mTOR signalling pathway, and MAPK signalling pathway to inhibit proliferation and promote apoptotic signal in the EC cells. The deregulation of the PI3K/Akt/mTOR pathway plays a fundamental role in breast cancer, such that the pharmacological suppression of PI3K/Akt/mTOR signalling showed inhibition of tumor growth in vivo [31]. Also, our previous study demonstrated that treatment with ProEGCG reduced tumour volume significantly in xenograft models of human breast cancer through proteasome inhibition and apoptosis induction [13]. Hence, it can be speculated that the ProEGCG would reduce tumour growth through proteasome inhibition on p38 MAPK/ERK signalling regulation to enhance autophagy and apoptosis.

By the same token, tumour growth was inhibited in the EC xenograft models subsequent to administration of ProEGCG. Based on the analysis of the lesion excised after ProEGCG treatment, a higher expression of apoptotic and necrotic activities was found (Fig. 6). Based on our microarray analysis, we found suppression of anti-apoptotic molecules, NOD1 and NAIP, in the cells treated with ProEGCG. As previously reported, both markers play central roles in anti-apoptotic activity and repression of tumour growth by inhibiting multiple caspase activities [32, 33]. And as reported in a previous literature, NOD1 was found to have tumour suppressor activity toward estrogen receptor (ER)-dependent breast cancer in a xenograft model [34]. Likewise, in ER-positive MCF-7 cells, NOD1 deficiency was found to be correlated with tumour growth, an increased sensitivity to estrogen-induced cell proliferation and impaired Nod1-dependent apoptosis. Similarly, the Nod1-dependent apoptosis was noted to be mediated by a caspase 8-cascade in an RIP2-dependent manner [32]. In another study, the proteomic profile of NOD-1 overexpressed cells suggests the involvement of several inflammation- and stress-related pathways (interconnected with the NF-κB, PI3K and MAPK cascades) toward the activation of protein degradation, cell cycle and cellular adhesion [35]. Similarly, the NLRB subfamily, namely the NLR family apoptosis inhibitory protein (NAIP), is an anti-apoptotic protein which acts by inhibiting (i) the activities of multiple caspases, including caspase − 3, caspase − 7 and caspase-9 [36], (ii) the autocleavage of procaspase − 9 and (iii) the cleavage of procaspase − 3 by caspase-9 [33]. Moreover, NAIP was shown to be a mediator of neuronal survival in several pathological conditions in preventing apoptosis [37]. Various studies showed that an increase in NAIP expression was associated with tumour growth [38, 39]. On the contrary, our study showed the capability of ProEGCG to suppress both anti-apoptotic molecules, NOD1 and NAIP.

On the contrary, our results showed a difference in sensitivity between these two endometrial cancer cell lines to ProEGCG. This might be attributed to the cells’ origin and properties. RL95–2 cells were derived from a stage II endometrial G2 adenosquamous carcinoma. This cell line with epithelial morphology is differentiated and steroid responsive. On the other hand, AN3CA cells were derived from serous type 2 endometrial carcinoma. This cell line is poorly differentiated, steroid receptor defective and tumorigenic. With the differences in histology, differentiation potential and hormone sensitivity, this should play a pivotal role toward the sensitivity toward ProEGCG in these two EC cell lines. As previously reported, metastatic tumours (e.g. AN3 CA) are highly resistant to cytotoxic agents. Hence, based on our results, although both cell lines showed a mitigation in AKT activation (Fig. 4), it was more pronounced in Rl95–2 cells than AN3 CA cells. As AKT is shown to control cell proliferation and response to apoptosis in multiple carcinoma, the downregulation in AKT activation would lead to greater inhibition of cell proliferation and increased apoptotic response in Rl95–2 cells than AN3 CA cells, as shown in Figs. 2 and 3.

Analogous to our previous report, the excised lesion also showed that the microvessel density in the EC xenograft models dwindled after ProEGCG treatment [16]. In regard to our previous publication [16], we can deduce that ProEGCG may induce apoptosis in the EC cells to prevent VEGFA secretion (in addition to preventing VEGFA from TAMs) and thus hinder vascularization produced by endothelial cells, and further promote EC cell death. Interestingly, these remarkable changes were not observed in EGCG treated cells. This clearly suggested the superiority of ProEGCG (than EGCG) with higher stability and bioavailability in reducing the growth of the EC tumor on the xenograft. Current anti-angiogenic drugs include VEGF inhibitor, VEGFR antibody and tyrosine kinase inhibitor [40]. Although some of these have yielded promising results in treating endometrial cancer, clinical trials are required to establish their safety and efficacy. Notably, unlike these anti-angiogenic drugs, ProEGCG did not produce any observable adverse effects (e.g. weight, renal or hepatic) in the xenograft bearing animals after receiving the treatment This suggests that the modification of the reactive hydroxyl groups with peracetate groups does not induce any toxicity. However, a longer longitudinal monitoring is warranted.

## Conclusion

In summary, we provide strong preclinical evidence of the benefit of ProEGCG as a potential therapeutic agent for EC. We showed that ProEGCG promoted cell death through inhibition of the Akt and ERK signalling pathways and subsequently downregulated the mechanistic pathway involved in cell proliferation and cell survival. The therapeutic benefit of ProEGCG permitted the shrinkage of lesion size concomitant with the attenuation of new vessel formation. Our results also showed metastatic serous cancers are not as responsive as endometrioid cancers. This may indicate a difference in response between metastatic cancer and cancer from which it originated. Most importantly, there was no side effect apparent in these treated animals. Moreover, our findings clearly support ProEGCG as a potential novel candidate for the treatment of endometrial cancer.

## Supplementary information

**Additional file 1 Supplementary Figure 1.** Effects of ProEGCG and EGCG on viability of normal human endometrial epithelial cells (HEECs). (A) HEECs were treated with increasing doses of EGCG (20, 40, and 60 μM) and ProEGCG (20, 40, and 60 μM) for 24, 48 and 72 h. Cell viability was assessed by MTT assay. The percentage of viable cells was calculated as the ratio of treated cells to the control cells. Data are presented as mean ± S.E.M. of three independent experiments. Significant differences from the control are indicated by * (*P* < 0.05) and ** (*P* < 0.01).

**Additional file 2 Supplementary Figure 2.** ProEGCG suppresses colony formation of endometrial cancer cells in 3D organoid co-culture. Endometrial cancer cells (AN3CA: 10,000 cells. RL95–2: 20,000 cells) were seeded onto matrigel. Cells were treated with either EGCG or ProEGCG (A) from day 2 to day 6, or (B) from day 7 to day 11. Photographs of spheroid sizes were taken under the microscope (100X), and the area of spheroids was measured. Data are presented as mean ± S.E.M. of three independent experiments. Significant differences from the control are indicated by * (*P* < 0.05) and ** (*P* < 0.01).

**Additional file 3 Supplementary Figure 3.** Immunofluorescence staining for detection of apoptosis in human endometrial carcinoma cell lines. (A) RL95–2 and AN3CA cells were treated with increasing doses of EGCG (20, 40, and 60 μM), or (B) ProEGCG (20, 40, and 60 μM) for 72 h with daily medium change and supplement. Localization and expression of Annexin-V (green) and propidium iodide (red) were analyzed by immunofluorescent staining. Nuclei were counterstained using DAPI (blue).

**Additional file 4 Supplementary Figure 4.** Positive Control of TUNEL staining. Mouse embryo E10 brain tissue sample was collected and incubated with a brief treatment of DNAse 1 after permeabilization. This was then proceeded with the TUNEL assay accordingly. Magnification 200x.

**Additional file 5 Supplementary Figure 5.** Full Western Blot Images from Figs. 3 and 4. Western blot analysis of the relative protein levels of Rl95–2 and AN3 CA cells. Quantitative analysis of the relative protein levels of Rl95–2 and AN3 CA cells was carried out in triplicate and repeated in 3 independent experiments. Rl95–2 and AN3 CA cells were probed with cleaved PARP, cleaved Caspase, GAPDH antibodies. Molecular weight (kDa) markers (MW) are shown: cleaved PARP is 116 kDa, cleaved Caspase-3 is 17/19 kDa, p-Akt is 60 kDa, Akt is 60 kDa, p-JNK is 54/46 kDa, JNK is 54/46 kDa, p-ERK is 44/42 kDa, ERK is 44/42 kDa, p-p38 is 43 kDa, p38 is 43 kDa, and GAPDH is 37 kDa.

## Data Availability

The datasets used and/or analysed during the current study are available from the corresponding author on reasonable request.

## Abbreviations

Akt: Protein kinase B
ALT: Alanine transaminase
ALP: Alkaline phosphatase
ATCC: American type culture collection
BAG1: BAG family molecular chaperone regulator 1
DAB: 3,3-Diaminobenzidine
DMSO: Dimethyl sulfoxide
ECC: Endometrial cancer cell
ECM: Extracellular matrix
EGCG: (−)-Epigallocatechin-3-gallate
ERK: Extracellular signal-regulated kinases
FITC: Fluorescein isothiocyanate
GAPDH: Glyceraldehyde 3-phosphate dehydrogenase
HEEC: Human endometrial epithelial cell
JNK: C-jun N-terminal kinases
MAPK: Mitogen-activated protein kinase
MTT: (3-(4,5-Dimethylthiazol-2-yl)-2,5-diphenyltetrazolium bromide)
NAIP: NLR family apoptosis inhibitory protein
NOD: Nucleotide-binding oligomerization domain-containing protein
PARP: Poly (ADP-ribose) polymerase
TUNEL: Terminal deoxynucleotidyl transferase-mediated dUTP nick end labeling
VEGF: Vascular endothelial growth factor
VEGF-R: Vascular endothelial growth factor receptor
