# Supplementary figures and images for: Therapeutic potential of a novel prodrug of green tea extract in induction of apoptosis via ERK/JNK and Akt signaling pathway in human endometrial cancer
Source: BMC Cancer. 2020 Oct 6;20:964. doi: 10.1186/s12885-020-07455-3 (PMC7539473; doi:10.1186/s12885-020-07455-3)

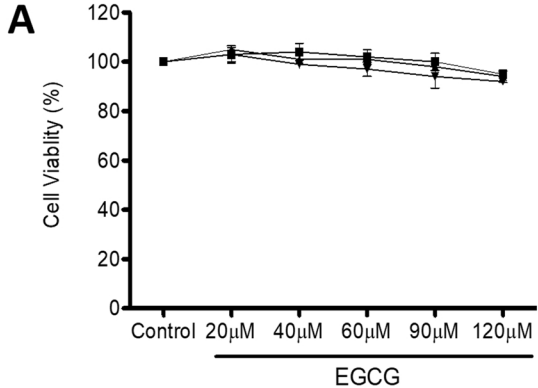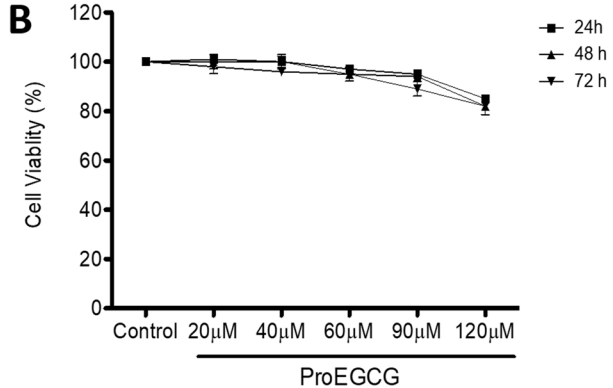

Supplement: Supplementary file 1 — Additional file 1 Supplementary Figure 1. Effects of ProEGCG and EGCG on viability of normal human endometrial epithelial cells (HEECs). (A) HEECs were treated with increasing doses of EGCG (20, 40, and 60 μM) and ProEGCG (20, 40, and 60 μM) for 24, 48 and 72 h. Cell viability was assessed by MTT assay. The percentage of viable cells was calculated as the ratio of treated cells to the control cells. Data are presented as mean ± S.E.M. of three independent experiments. Significant differences from the control are indicated by * (P < 0.05) and ** (P < 0.01). [file 12885_2020_7455_MOESM1_ESM.pdf]

## A Treatment from Day 2 - Day 6

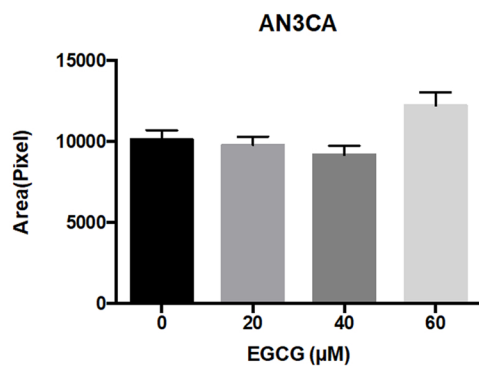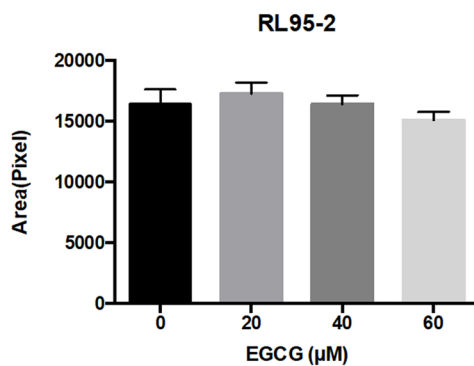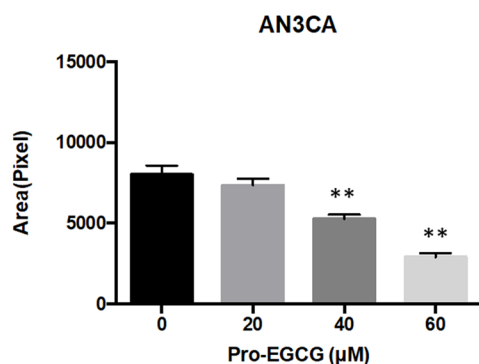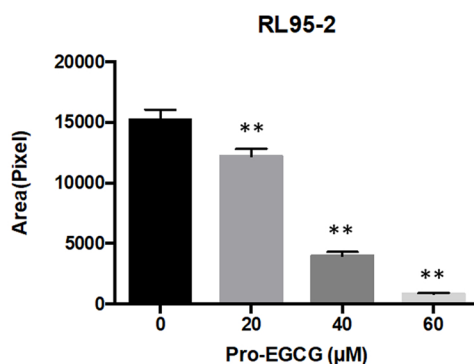

## B Treatment from Day 7 - Day 11

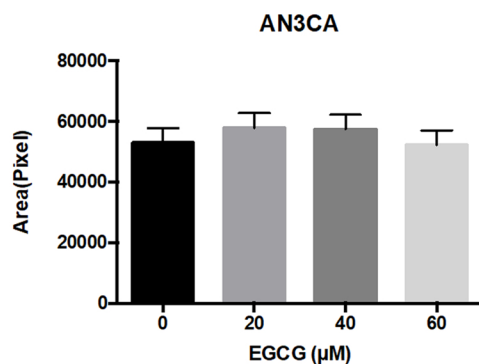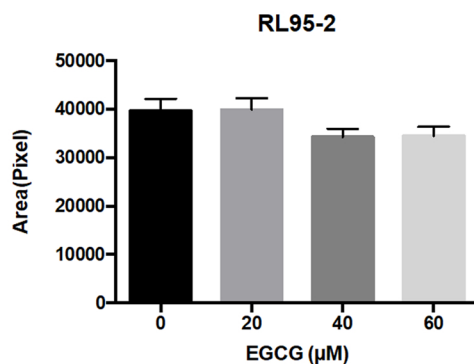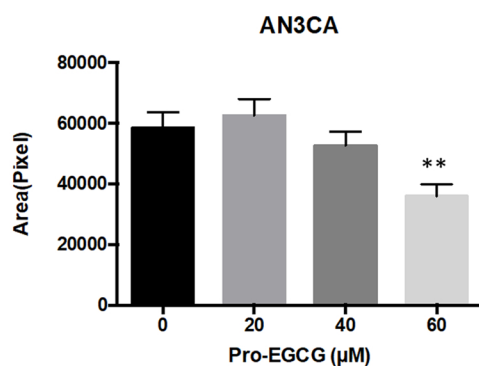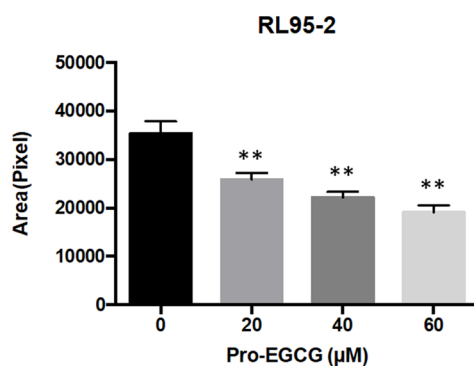

Supplement: Supplementary file 2 — Additional file 2 Supplementary Figure 2. ProEGCG suppresses colony formation of endometrial cancer cells in 3D organoid co-culture. Endometrial cancer cells (AN3CA: 10,000 cells. RL95–2: 20,000 cells) were seeded onto matrigel. Cells were treated with either EGCG or ProEGCG (A) from day 2 to day 6, or (B) from day 7 to day 11. Photographs of spheroid sizes were taken under the microscope (100X), and the area of spheroids was measured. Data are presented as mean ± S.E.M. of three independent experiments. Significant differences from the control are indicated by * (P < 0.05) and ** (P < 0.01). [file 12885_2020_7455_MOESM2_ESM.pdf]

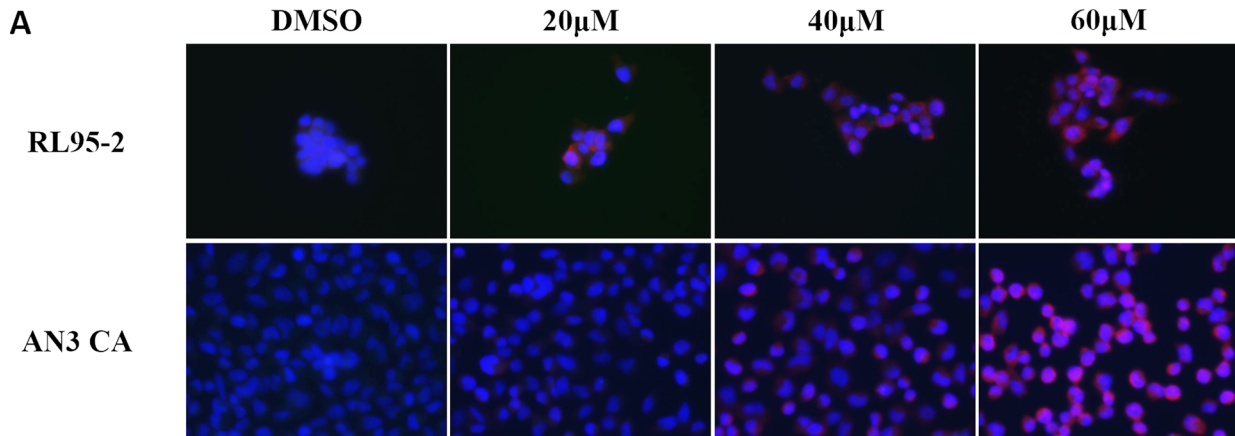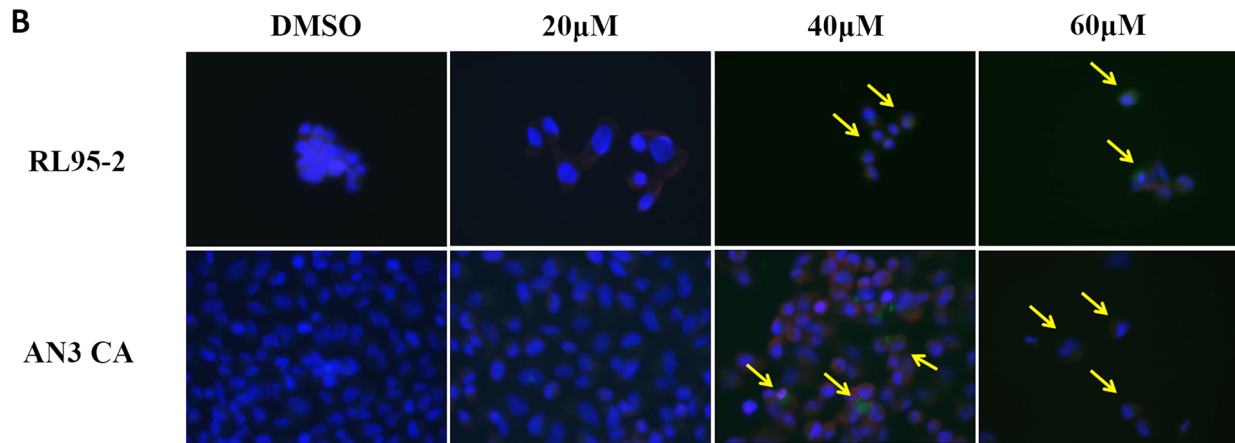

Supplement: Supplementary file 3 — Additional file 3 Supplementary Figure 3. Immunofluorescence staining for detection of apoptosis in human endometrial carcinoma cell lines. (A) RL95–2 and AN3CA cells were treated with increasing doses of EGCG (20, 40, and 60 μM), or (B) ProEGCG (20, 40, and 60 μM) for 72 h with daily medium change and supplement. Localization and expression of Annexin-V (green) and propidium iodide (red) were analyzed by immunofluorescent staining. Nuclei were counterstained using DAPI (blue). [file 12885_2020_7455_MOESM3_ESM.pdf]

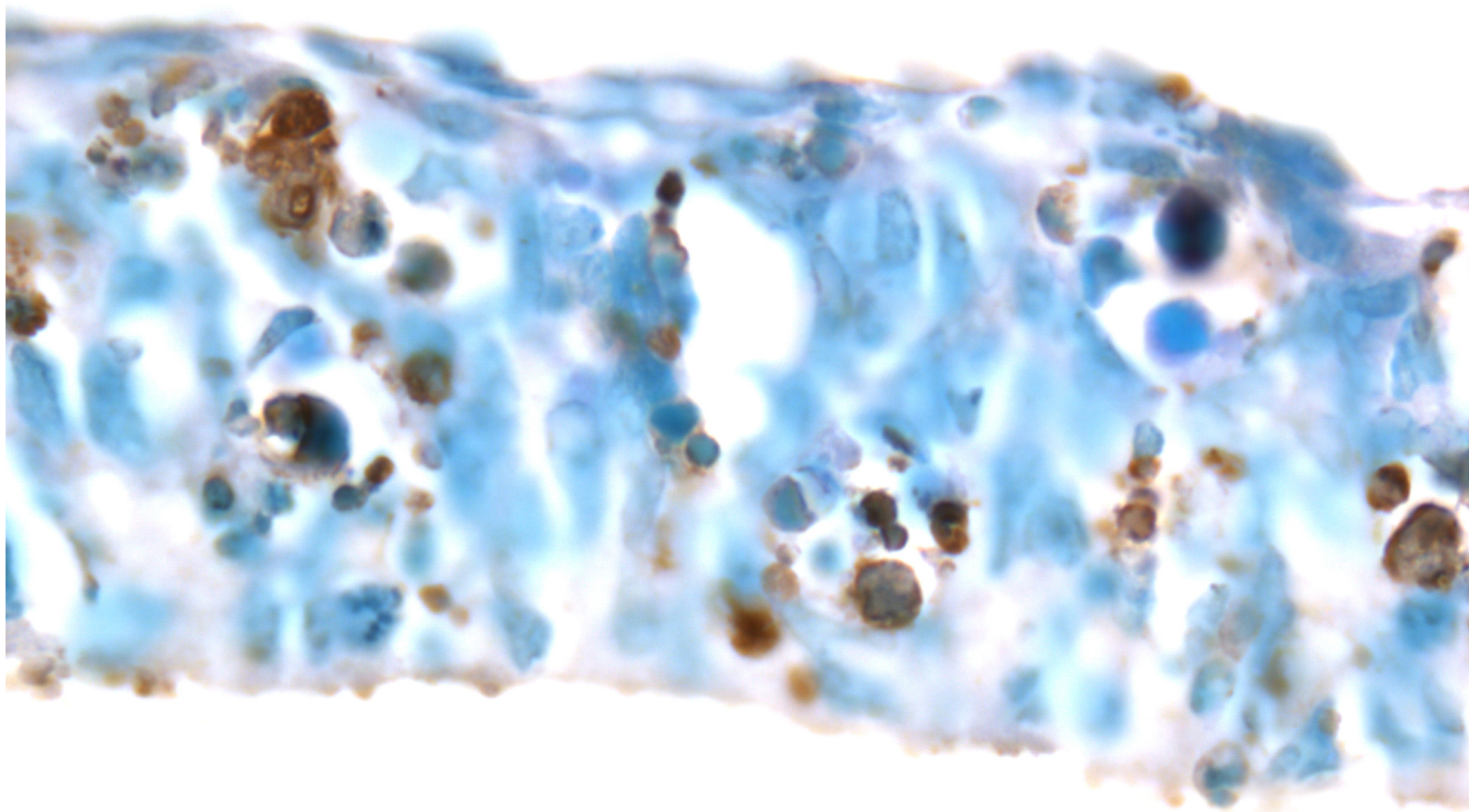

Supplement: Supplementary file 4 — Additional file 4 Supplementary Figure 4. Positive Control of TUNEL staining. Mouse embryo E10 brain tissue sample was collected and incubated with a brief treatment of DNAse 1 after permeabilization. This was then proceeded with the TUNEL assay accordingly. Magnification 200x. [file 12885_2020_7455_MOESM4_ESM.pdf]
