# Supplementary material for: Therapeutic potential of a novel prodrug of green tea extract in induction of apoptosis via ERK/JNK and Akt signaling pathway in human endometrial cancer
Source: BMC Cancer. 2020 Oct 6;20:964. doi: 10.1186/s12885-020-07455-3 (PMC7539473; doi:10.1186/s12885-020-07455-3)

## Figure 3E

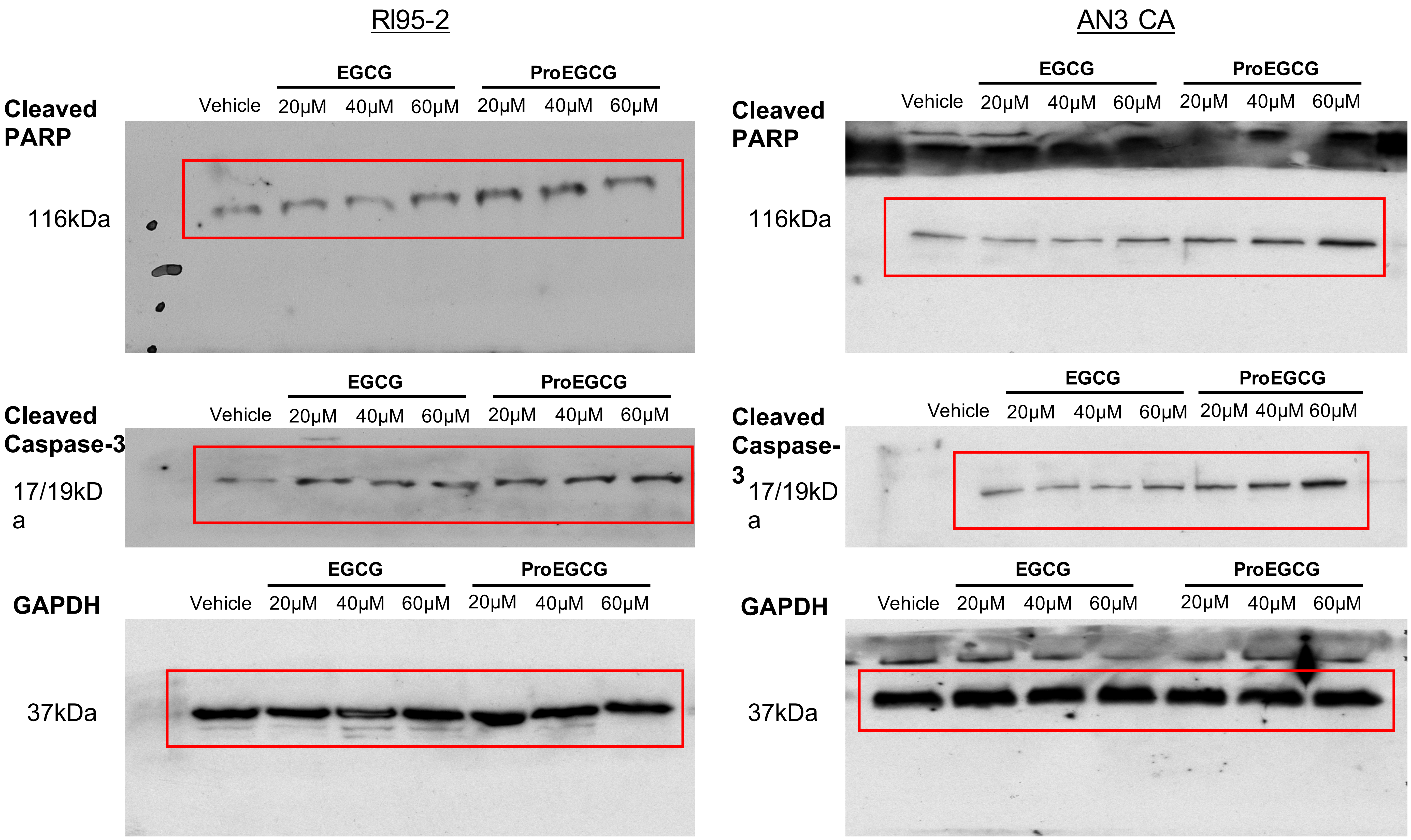
RI95-2
AN3 CA
EGCG
ProEGCG
Vehicle
20µM
40µM
60µM
20µM
40µM
60µM
Cleaved PARP
116kDa
Cleaved Caspase-3
17/19kDa
GAPDH
37kDa

Figure 4A

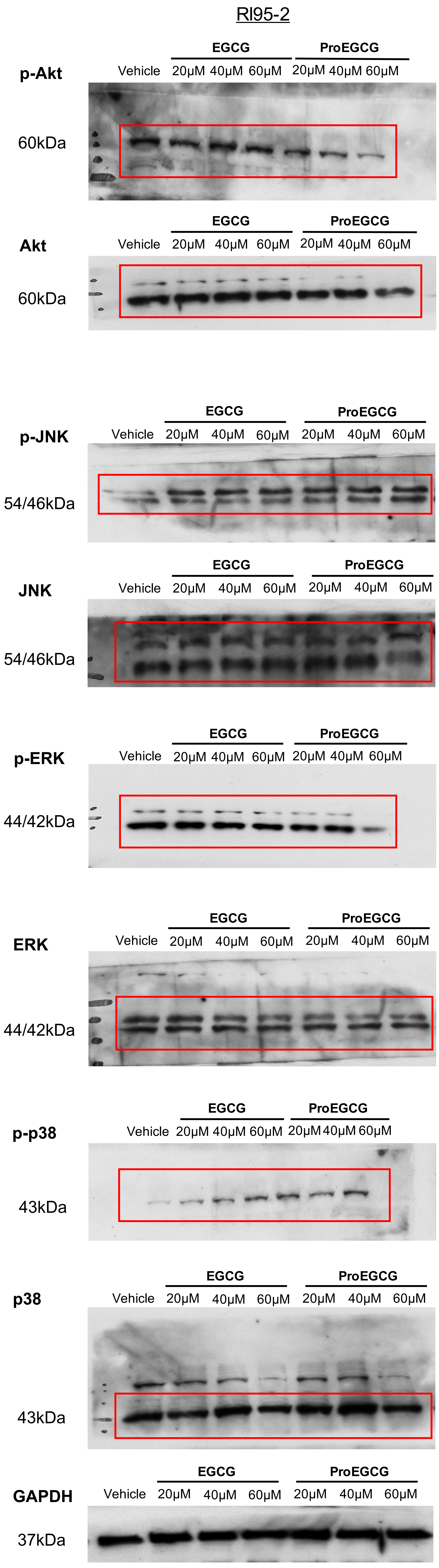


Figure 4B

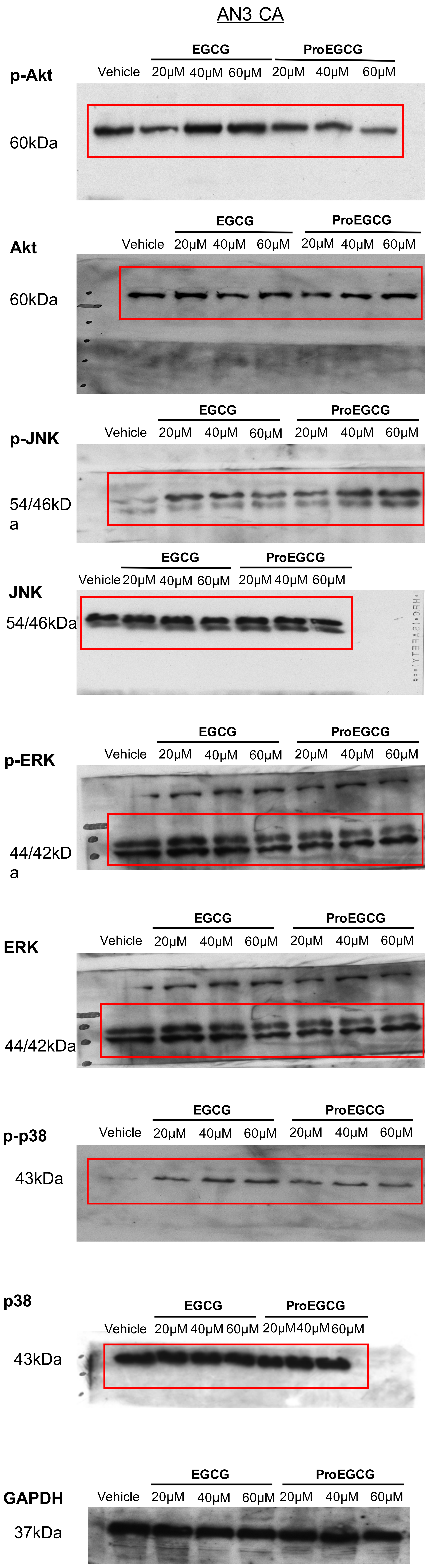

Supplement: Supplementary file 5 — Additional file 5 Supplementary Figure 5. Full Western Blot Images from Figs. 3 and 4. Western blot analysis of the relative protein levels of Rl95–2 and AN3 CA cells. Quantitative analysis of the relative protein levels of Rl95–2 and AN3 CA cells was carried out in triplicate and repeated in 3 independent experiments. Rl95–2 and AN3 CA cells were probed with cleaved PARP, cleaved Caspase, GAPDH antibodies. Molecular weight (kDa) markers (MW) are shown: cleaved PARP is 116 kDa, cleaved Caspase-3 is 17/19 kDa, p-Akt is 60 kDa, Akt is 60 kDa, p-JNK is 54/46 kDa, JNK is 54/46 kDa, p-ERK is 44/42 kDa, ERK is 44/42 kDa, p-p38 is 43 kDa, p38 is 43 kDa, and GAPDH is 37 kDa. [file 12885_2020_7455_MOESM5_ESM.pdf]
